# Human platelets repurposed as vehicles for *in vivo* imaging of myeloma xenotransplants

**DOI:** 10.18632/oncotarget.8517

**Published:** 2016-03-31

**Authors:** Lu Dai, Ning Gu, Bao-An Chen, Gerard Marriott

**Affiliations:** ^1^ Department of Hematology, Zhongda Hospital, Medical School, Southeast University, Nanjing, China; ^2^ School of Biological Science and Medical Engineering, Southeast University, Nanjing, China; ^3^ Department of Bioengineering, University of California, Berkeley, CA, United States of America

**Keywords:** human platelets, repurposing platelets, tumor-targeting, in vivo imaging, myeloma xenotransplants

## Abstract

Human platelets were identified in tumors by Trousseau in 1865, although their roles in tumor microenvironments have only recently attracted the attention of cancer researchers. In this study we exploit and enhance platelet interactions in tumor microenvironments by introducing tumor-targeting and imaging functions. The first step in repurposing human platelets as vehicles for tumor-targeting was to inhibit platelet-aggregation by cytoplasmic-loading of kabiramide (KabC), a potent inhibitor of actin polymerization and membrane protrusion. KabC-Platelets can accumulate high levels of other membrane-permeable cytoxins and probes, including epidoxorubicin, carboxyfluorescein di-ester and chlorin-e6. Finally, mild reaction conditions were developed to couple tumor-targeting proteins and antibodies to KabC-platelets. Fluorescence microscopy studies showed KabC-platelets, surface-coupled with transferrin and Cy5, bind specifically to RPMI8226 and K562 cells, both of which over-express the transferrin receptor. Repurposed platelets circulate for upto 9-days a feature that increases their chance of interacting with target cells. KabC-platelets, surface-coupled with transferrin and Cy7, or chlorin-e6, and injected in immuno-compromised mice were shown to accumulate specifically in sub-cutaneous and intra-cranial myeloma xenotransplants. The high-contrast, *in vivo* fluorescence images recorded from repurposed platelets within early-stage myeloma is a consequence in part of their large size (φ∼2μm), which allows them to transport 100 to 1000-times more targeting-protein and probe molecules respectively. Human platelets can be configured with a plurality of therapeutic and targeting antibodies to help stage tumor environments for an immunotherapy, or with combinations of therapeutic antibodies and therapeutic agents to target and treat cardiovascular and neurologic diseases.

## INTRODUCTION

Platelets are generated from cytomegakaryocytes as discoid shaped, closed membrane, anucleate cytoplasmic fragments with a diameter of ∼2μm. Human platelets circulate in the vascular system for about 9-days where they have essential roles in hemostasis and inflammation [1-4]. A typical adult contains ∼4×10^12^ platelets, and each day, ∼10^8^ older platelets are removed and ∼10^8^ new platelets are produced by cytomegakaryocytes [3]. While primarily confined to the blood, platelets can gain access to the tumor microenvironment *via* passive diffusion across leaky capillaries, or through associations with neutrophils and other immune cells [1, 2, 4]. We reasoned it should be possible to exploit and to further enhance this privileged access to tumor microenvironments by *ex vivo* engineering human platelets with tumor-targeting proteins, imaging probes and cytotoxins. Repurposed platelets represent a new class of living vehicle for *in vivo* imaging, and targeted-delivery of protein therapeutics or small molecule cytotoxins to tumors. We have developed simple protocols to repurpose human platelets for these roles that involve: (a), inhibiting platelet aggregation; (b), loading the platelet cytosol with detection probes for *in vivo* imaging; (c), loading the platelet cytosol with cytotoxins for drug delivery; (d), linking targeting antibodies or protein ligands to the platelet surface for tumor targeting. Repurposed platelets have several advantages over artificial nanoparticles for *in vivo* targeting and imaging of tumor cells. First, platelets are recognized as self by the host, and they are widely transfused into patients as part of a cancer therapy [5]. Second, platelets have privileged access to tumor microenvironments where they may interact with tumor cells and immune cells [1, 4]. Third, platelets are cleared exclusively in the liver or spleen after an average circulation time of 9-days [1], whereas nanoparticle-derived vehicles [6] are typically cleared within 3∼5 hours by macrophages and the liver [7]. This short circulation time reduces the chance for encounters with tumor cells, and especially those deep in the tumor. On the other hand, the longer circulation of injected platelets increases their chance of interacting with target tumor cells. Moreover, the rapid removal of nanoparticles loaded with cytotoxins would expose the liver to high levels of cytotoxin that could trigger liver damage. Interestingly, a recently described approach to lengthen the circulation time of injected nanoparticles involves cloaking the nanoparticle surface with fragments of platelet membranes [8]. Fourth, human platelets are much larger than most nanoparticles (φ ~ 2 μm *vs* < 0.2 μm, respectively), and in principle they can accommodate many more surface-coupled targeting proteins, and internalized probes (∼100-fold and 1000-fold respectively).

Platelet-activation is characterized by the formation of numerous actin polymerization-driven membrane protrusions that promote platelet-aggregation and clumping [5, 9]. Platelet-aggregation prevents their application as stand-alone vehicles for tumor-targeting. We have developed simple approaches to suppress both specific and non-specific platelet-aggregation, one of which involves loading platelets with kabiramide C (KabC), a natural product membrane permeable drug that binds tightly to the barbed-end of the actin filament where it effectively inhibits actin polymerization [10-12]. KabC-loaded platelets do not produce membrane protrusions or aggregate on exposure to thrombin, or during the physical manipulations used during their transformation to tumor-targeting vehicles.

KabC-platelets were configured with one or more membrane permeable drugs and detection probes, including epidoxorubicin, chlorin e6 and carboxyfluorescein diester (CFDA). They were subsequently chemically-coupled on their surface with tumor-targeting proteins, including maleimide conjugates of transferrin and antibodies. Near infra-red (NIR) fluorophores used for *in vitro* and *in vivo* imaging were introduced either by chemical-coupling of Cy5-NHS or Cy7-NHS to the surface, or else by loading the cytoplasm with a membrane permeable NIR-fluorophore. Confocal fluorescence microscopy and fluorescence-activated cell sorting (FACS) techniques were used to show KabC-platelets, surface-coupled with human transferrin and Cy5, bind specifically to RPMI8226 multiple myeloma cells and K562 leukemia cells, both of which over-express the transferrin receptor [13]. KabC-platelets, surface-coupled with transferrin and Cy7, were shown using *in vivo* fluorescence imaging to accumulate within RPMI8226 cell-derived myeloma xenotransplants in immuno-compromised mice [14-17]. Our studies also suggest the possibility of engineering human platelets to target other diseased states in part by introducing surface-coupled antibodies against biomarkers unique to the diseased cell.

## RESULTS

### Suppressing specific and non-specific aggregation of human platelets

**Figure 1:**
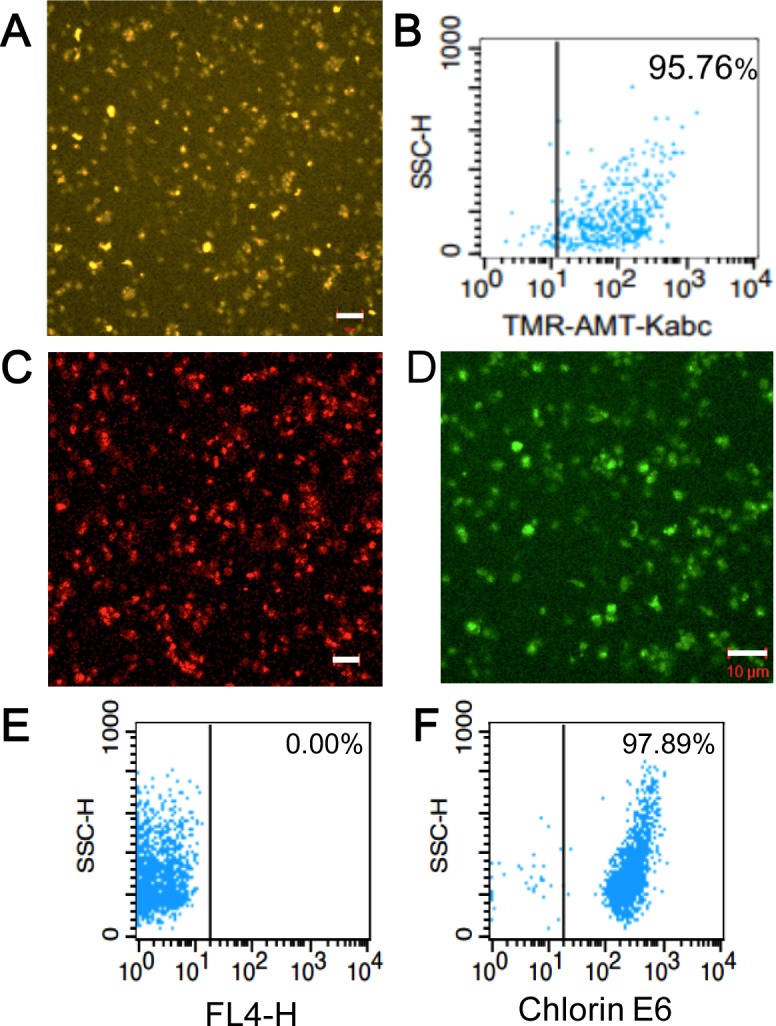
Characterization and analysis of tumor-targeting platelets **A.** Confocal fluorescence image showing the accumulation of TMR-KabC in a field of human platelets. Bar = 10 μm. **B.** FACS analysis of TMR-KabC loading of purified human platelets recorded 24-hours after incubating with 10^8^ platelets/ml with TMR-KabC at 5 μM. **C.** Confocal fluorescence image of the intrinsic fluorescence of EPI in KabC-platelets. Bar = 10 μm. **D.** Confocal fluorescence image of KabC-platelets loaded with CFDA. CFDA is de-esterified within the platelet to produce a fluorescent di-anionic fluorescein probe that is trapped in the cytosol. Measure bar = 10 μm. **E.** FACS analysis of KabC-platelets without any probe labeling. **F.** FACS analysis of KabC-platelets loaded with a 30μM solution of chlorin-e6 in KabC-platelets and recorded 24-hours later.

**Figure 2:**
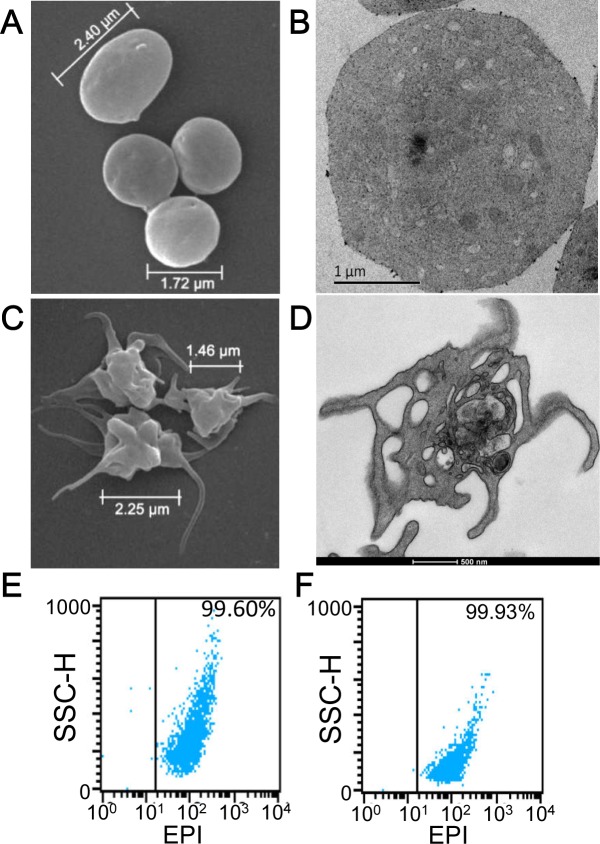
Characterization of KabC-platelets **A.** SEM image of a KabC-platelets previously exposed to thrombin (1U/ml). **B.** TEM image of a single KabC-platelet previously exposed to thrombin (1U/ml). **C.** SEM image of human platelets without KabC previously exposed to 1 U/ml thrombin. **D.** TEM image of a single human platelet without KabC previously exposed to 1 U/ml thrombin. FACS analysis of light-scattering for KabC-platelet loaded with EPI **E.** before, and **F.** after an exposure to 1U/ml thrombin.

An essential step in repurposing human platelets as vehicles for tumor-targeting is to overcome their tendency to aggregate and clump. Platelet aggregation was inhibited by the passive loading of freshly acquired, out-dated human platelets with KabC or tetramethylrhodamine-KabC (TMR-KabC) [10, 11]. These drugs block platelet aggregation by inhibiting actin polymerization and associated formation of membrane protrusions that are characteristic of platelet-activation and aggregation [9]. Confocal fluorescence images of human platelets incubated with TMR-KabC show the drug accumulates in the cytosol where it produces a strong emission that allows for high-contrast imaging of individual platelets (Figure 1A). The concentration and incubation-time dependence of TMR-KabC and KabC-loading of platelets was investigated further by recording the intensity of TMR-fluorescence in platelets (Supplementary Figure 1). The intensity of TMR-fluorescence in platelets increased as a function of the incubation time, and it did not saturate over the concentration range of TMR-KabC used in this study (0∼5 μM), which would suggest platelets could accumulate an even higher concentration of the probe. The final (standard) condition that emerged from these analyses was to incubate ∼10^8^ platelets with KabC (5μM) in mHBSS for 15-minutes at 22°C, followed by low-speed centrifugation and washing with mHBSS. The standard KabC-loading condition resulted in 95.8% of platelets having a TMR-fluorescence signal that exceeded the highest fluorescence signal recorded for untreated platelets (Figure 1B). The finding that platelets sequester TMR-KabC (and KabC) in their cytosol was expected, as both drugs are cell permeable and are effectively trapped in the platelet *via* their interactions with actin (∼400 μM, 9-12]. Proof that TMR-KabC and KabC were effective in suppressing thrombin-mediated platelet aggregation was provided by scanning electron microscopy (SEM) and transmission electron microscopy (TEM) analyses of thrombin-treated platelets loaded with, or without KabC (Figure 2A, 2B and 2C, 2D, respectively). Platelets loaded with KabC that were exposed to thrombin maintained the characteristic smooth and spherical shape of the non-aggregated platelet (Figure 2A, 2B), whereas platelets lacking KabC and treated with thrombin were irregular in shape and displayed numerous long membrane protrusions that are characteristic of activated and aggregated platelets (Figure 2C, 2D).

### Loading platelets with cytotoxins, NIR-fluorophores and photodynamic therapy probes

Having successfully inhibited platelet-aggregation with KabC, we used confocal fluorescence microscopy and FACS analyses to optimize the experimental conditions used to load KabC-platelets with other membrane permeable drugs and detection probes. These studies showed platelets accumulated high levels of epidoxorubicin (EPI), a potent and intrinsically fluorescent cytotoxin (Figure 1C), CFDA, a fluorogenic probe suitable for *in vitro* fluorescence imaging of platelets (Figure 1D), and chlorin-e6, a dual-purpose, red-emitting fluorophore and photodynamic probe (Figure 1E, 1F [18]). The fluorescence signals of platelets loaded with chlorin-e6 platelets exceeded the autofluorescence signal of unlabeled platelets by a wide margin (Figure 1E, 1F, vertical lines indicate the background intensity threshold of unlabeled platelets). The strong fluorescence of CFDA and chlorin-e6 within platelets allowed us to record high-contrast images of individual platelets during a single scan of the field using a low power of the 488nm or 639 nm light source, respectively. FACS analysis also showed that KabC-platelets retained an internalized drug or probe for at least 24-hours (Figure 1B, 1F). Finally, FACS analysis of the intrinsic fluorescence of EPI within KabC-platelets before and after treatment with thrombin did not reveal any loss of EPI, which suggests the membrane was not compromised by thrombin-treatment (99.6% *versus* 99.9%, before and after thrombin treatment respectively; Figure 2E, 2F).

### Surface-coupling platelets with targeting proteins and NIR fluorescent probes

**Figure 3:**
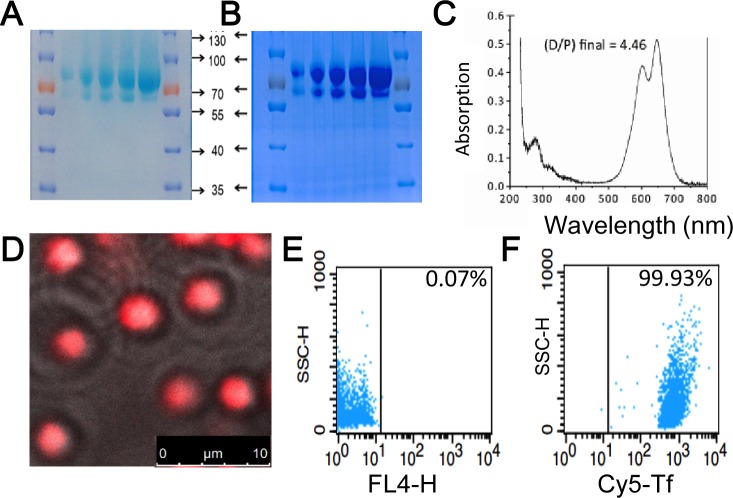
Characterization of transferrin conjugates and their coupling to KabC-platelets SDS-PAGE images of transferrin labeled with Cy5 and PDM showing **A.** the unstained gel with the light blue color originating from absorption of transferrin linked Cy5-probes, and **B.** the same gel stained with Coumassie Blue. Cy5-labeled transferrin shows up as band at ∼80kD in both cases. **C.** Absorption spectrum of the transferrin of Cy5/PDM. Analysis of the absorption data at 650nm (Cy5) and 280 nm (transferrin) is used to calculate a labeling ratio of 4.5 Cy5 molecules per transferrin molecule. **D.** Overlay of the phase-contrast and Cy5-fluorescence images of KabC-platelets coupled with transferrin conjugated to Cy5/PDM. Bar = 10 μm **E.** FACS analysis of the background fluorescence of KabC-platelets measured in the Cy5-emission channel. **F.** FACS analysis of the optimized loading condition developed to couple transferrin conjugated to Cy5/PDM to thiol-containing KabC-platelets measured in the Cy5-emission channel.

Mild reaction conditions were developed to link human transferrin and tumor-targeting antibodies to the outer membrane of KabC-platelets. Transferrin was chosen to target RPMI8226 multiple myeloma and K562 leukemia as previous studies have shown these cells over-express the transferrin receptor [13]. First, a 5mg/ml solution of human transferrin (Sigma) was mixed with an excess of 2-iminothiolane (Traut's reagent) in de-aerated PBS to generate thiol groups on the protein [19]. The thiolated transferrin conjugate was purified using PD-10 chromatography in PBS, and subsequently mixed with an excess of N,N'-(1,4-phenylene)-dimaleimide (PDM) to generate pendant maleimide groups (Transferrin-PDM) [20]. In some studies (Figure 3D, 3F and Supplementary Figure 2), transferrin-PDM was reacted with Cy5-NHS or Cy7-NGS in PBS followed by purification using PD-10 chromatography. Transferrin conjugates bearing Cy5 and maleimide groups were concentrated and stored in 100 μL aliquots at −80°C. Proof that Cy5 probes were chemically-coupled to transferrin-PDM was provided by SDS-PAGE analysis of the transferrin conjugate. The transferrin band (∼80kD) was deliberately over-loaded in order to visualize the Cy5-labeled (blue-colored) 80kD protein along with its proteolysed fragments (Figure 3A, 3B). Absorption spectrometric analysis of the purified Cy5-transferrin conjugate revealed an average labeling ration of ∼4.5 Cy5 molecules (Cy5/transferrin; Figure 3C). The blue-shifted shoulder in the Cy5-absorption spectrum results from molecular interactions between Cy5 molecules on the transferrin molecule (Figure 3C; [21]).

Next, we developed a simple protocol to couple the doubly-labeled transferrin conjugate (Cy5/PDM) to the surface of KabC-platelets. KabC-platelets were first incubated with 2-iminothiolane (Traut's reagent) for 15-minutes in de-aerated buffer to generate thiol groups on their outer membrane. These platelets were washed with de-aerated buffer, centrifuged at low speed, and re-suspended in a PBS solution containing 0.2mg/ml solution of doubly-labeled transferrin conjugate for 15-minutes followed by centrifugation and re-suspension in mHBBS. Chemical-coupling of transferrin-PDM/Cy5 conjugates to KabC-platelets was confirmed using confocal fluorescence microscope imaging (Figure 3D) and FACS analysis (Figure 3E, 3F and Supplementary Figure 2). A representative overlap image (bright-field and Cy5-fluorescence; Figure 3D) shows non-aggregated and spherical platelets that are uniformly labeled with Cy5. FACS analysis of a larger population of the KabC-platelets surface-coupled with Cy5/PDM-transferrin show more than 99.9% have a Cy5-fluorescence signal that exceeds the highest background signal of unlabeled platelets (Figure 3F and 3E respectively, with the vertical lines indicating the background threshold; Supplementary Figure 2). A high level of Cy7-transferrin labeling of KabC-platelets is also evident from the blue-colored, gravity-sedimented suspension of Cy7-coupled KabC-platelets (Supplementary Figure 2).

**Figure 4:**
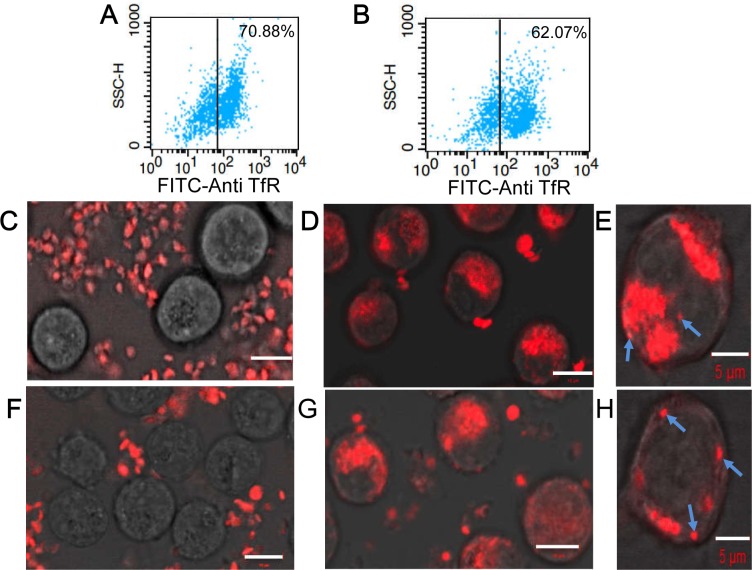
Characterization of interactions between KabC-platelets and tumor cells **A.** FACS analysis of RPMI8226 cells stained directly with a FITC-conjugated antibody against human transferrin receptor. **B.** FACS analysis of K562 cells stained directly with a FITC-conjugated antibody against human transferrin receptor. **C.** Confocal Cy5-fluorescence image of surface-attached RPMI 8226 cells recorded 8-hours after being incubated with Cy5-coupled KabC-platelets. Bar = 10 μm **D.** Confocal Cy5-fluorescence image of surface-attached RPMI 8226 cells recorded 8-hours after being incubated with KabC-platelets coupled with Cy5 and transferrin. Bar = 10 μm **E.** Higher-resolution confocal Cy5-fluorescence image of surface-attached RPMI8226 cells recorded 8-hours after being incubated with KabC-platelets coupled with Cy5 and transferrin showing individual surface attached platelets and clusters of Cy5-fluorescence (cyan arrows). Bar = 5 μm **F.** Confocal Cy5-fluorescence image of surface-attached K562 cells recorded 8-hours after being incubated with Cy5-coupled KabC-platelets. Bar = 10 μm **G.** Confocal Cy5-fluorescence image of surface-attached K562 cells recorded 8-hours after being incubated with KabC-platelets coupled with Cy5 and transferrin. Bar = 10 μm **H.** Higher-resolution confocal Cy5-fluorescence image of surface-attached K562 cells recorded 8-hours after being incubated with KabC-platelets coupled with Cy5 and transferrin showing individual surface attached platelets and clusters of Cy5-fluorescence (cyan arrows). Bar = 5 μm

**Figure 5:**
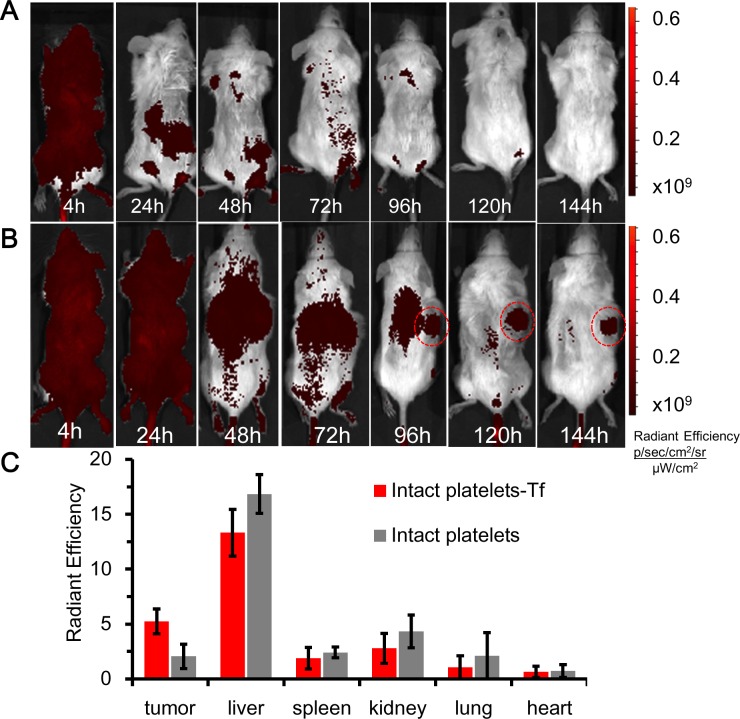
*in vivo* NIR-fluorescence imaging of platelets in mice bearing myeloma xenotransplants **A.** Time course of Cy7-fluorescence images of a mouse bearing a sub-cutaneous RPMI8226 xenograft on its back injected with Cy7 coupled KabC-platelets on day-15. The look up tables presented have units of radiant efficiency (photons.sec^−1^.cm^−2^.steradian^−1^) / μW.cm^2^. **B.** Time course of the distributions of Cy7-fluorescence in a mouse bearing a sub-cutaneous RPMI8226 cell xenotransplant on its back, and injected with Cy7/transferrin coupled platelets on day-15. **C.** Bar graph showing the integrated intensities of Cy7-fluorescence in select organs excised from mice injected with Cy7-coupled platelets with transferrin (test) or without transferrin (control).

Two other protocols were developed to prepare KabC-platelets surface-coupled with transferrin and Cy5 or Cy7. In one protocol thiolated platelets were first coupled with a transferrin-PDM conjugate, and the platelets reacted separately with 0.2 mg/ml of Cy5-NHS or Cy7-NHS. The Cy5 or Cy7-coupled KabC-platelets prepared using this protocol were used for studies described in Figures 4 and 5, and Supplementary Figure 3. In the second protocol thiolated KabC-platelets were reacted with transferrin that had been doubly-labeled with Cy5-NHS and maleimidobenzoic acid succinimide acid (MBS). This latter protocol was used to prepare KabC-platelets with a full cargo of cytotoxins, detection probes and surface-coupled targeting proteins within 60-minutes of receiving out-dated platelets. Repurposed platelets were stable in mHBBS for up to 7-days - for example, the thrombin treated KabC-platelets shown in Figure 2A had been stored for 7-days at 4^o^ in platelet stabilizing buffer before being prepared for imaging in the electron microscope.

### *In vitro* imaging of interactions between repurposed platelets and human tumor cells

Next we used confocal fluorescence microscopy and FACS analysis to quantify interactions between Cy5/transferrin-coupled KabC-platelets and RPMI8226 cells and K562 cells. We confirmed each of these cell lines over-express the transferrin receptor [13] by incubating cells with a FITC-labeled primary antibody against the human transferrin receptor (Figure 4A, 4B) followed by FACS analysis - more than 70% of RPMI8226 cells, and > 62% of K562 cells exhibited a FITC-fluorescence signal that exceeded the maximum background signal of unlabeled cells (indicated by the vertical lines in Figure 4A, 4B). Fluorescence images of KabC-platelets coupled with Cy5 alone, or Cy5 and transferrin were recorded after an 8-hour incubation with RPMI8226 cells (Figure 4C, 4D, 4E), or K562 cells (Figure 4F, 4G, 4H). The overlap of the Cy5-fluorescence and bright-field images for control platelets (Cy5-coupled KabC-platelets without transferrin) did not reveal any significant binding of platelets to RPMI8226 cells or K562 cells (Figure 4C and 4F respectively). On the other hand, a significant number of KabC-platelets surface-coupled with transferrin and Cy5 were found on RPMI8226 cells or K562 cells (Figure 4D, 4E and 4G, 4H respectively). These studies suggest that repurposed platelets bind specifically to RPMI8226 cells, and K562 cells by interactions between transferrin molecules on the platelet membrane, and the large number of transferrin receptors on the surface of these cells. The Cy5-fluoresence on RPMI8226 cells and K562 cells remained spotty even after a long period of incubation, which might indicate the large diameter of bound platelets prevents their internalization by endocytosis [22].

### *In vivo* imaging of tumor-targeting platelets

Next we characterized the *in vivo* distributions of KabC-platelets surface-coupled with Cy7 and transferrin, or platelets without transferrin in immuno-compromised mice. RPMI8226 cells were injected sub-cutaneously on the back of NOD/SCID mice [14-16]. Myeloma xenotransplants were visible in mice around 2-4 weeks after injecting RPMI8226 cells. RPMI8226 cells were used for these studies as they over-express the transferrin receptor [13]. Moreover, previous studies have employed sub-cutaneous myeloma xenotransplants derived from injections of RPMI8226 cells to study the effectiveness of antibody-based tumor therapies [14-17]. NOD/SCID mice were used as hosts for the myeloma xenotransplant described in our studies - animals homozygous for the SCID mutation have impaired T and B cell lymphocyte development, while the NOD background results in deficient natural killer (NK) cell function. Myeloma xenotransplants grow more efficiently in NOD/SCID mice as a result of their immune deficiency, which facilitates the growth of sub-cutaneous and intra-cranial injected RPMI8226 cells. The average volume of xenotransplants that developed after subcutaneous injection of RPMI8226 cells reached 80∼100 mm^3^ after 7-10 days, and increased to ∼2000 mm^3^ by ∼21 days.

A Caliper NIR-fluorescence imaging system was used to image and analyze the distributions of repurposed platelets injected into mice. Sub-cutaneous xenotransplants were allowed to develop to ∼100mm^3^ in test mice. Test platelets (transferrin) and control platelets (no transferrin) were injected separately into the tail veins of NOD/SCID mice (*n* = 3 per group), and whole body Cy7-fluorescence images recorded over a period of 6-days. Control and test platelets were found throughout the body of injected mice during the first 24-hours, as evidenced by the strong and uniform Cy7-fluorescence in representative mice shown in Figure 5A, 5B. The accumulation of test platelets in a representative sub-cutaneous xenotransplant was evident about 96-hours after platelet-injection, while image contrast improved beyond that time owing to the clearance of unbound and aged platelets (Figure 5B at 144 hours). The targeting of repurposed platelets to myeloma xenotransplants was further evaluated by recording Cy7-fluorescence from the organs excised from mice previously injected with test or control platelets (Figure 5C). The area-normalized Cy7-fluorescence signals from excised tumors was 2.54 times higher for Cy7/transferrin-coupled KabC-platelets compared to Cy7-coupled KabC-platelets without transferrin (5.25+/−1.11 *vs* 2.07+/−1.12 (photons /s/cm^2^/sr)/(μW/cm^2^) respectively). The discovery of a weak Cy7-fluorescence signal in the excised tumors of mice injected with control platelets was not unexpected, as previous studies have shown activated-platelets do localize to tumors [1, 3, 4]. Cy7-fluorescence was also evident in the excised livers of mice injected with test or control platelets (13.32+/−2.13 and 16.85+/−1.78 (photons /s/cm^2^/sr)/(μW/cm^2^) respectively), and in the spleens of mice injected with test and control platelets (1.89+/−0.99 and 2.41+/−0.40 (photons/s/cm^2^/sr)/(μW/cm^2^) respectively). Platelet accumulation in these organs was also expected as aged-platelets are known to be cleared by Kupfer cells. Interestingly, high levels of Cy7-fluorescence were also found in the kidneys of both groups of mice ([2.79+/−1.37 and 4.33+/−1.49 (photons/s/cm^2^/sr)/(μW/cm^2^] respectively), and in their urine. Given that most if not all chemical modifications of Cy7, including oxidation and fragmentation, result in a loss of far-red absorption and fluorescence (λ_ex_ ∼650nm, λ_ex_ ∼670nm), the finding of significant Cy7-fluorescence in the urine of injected mice would suggest Cy7 is not modified during its residence in the mouse. The effectiveness of test platelets in targeting myeloma xenotransplants was further evaluated by calculating the ratio of Cy7-fluorescence among excised organs (myeloma xenotransplant, liver, spleen and kidney). The ratios for the tumor/liver, tumor/spleen and tumor/kidney in the test and control mice were consistently higher for test platelets compared to control platelets, and calculated as 0.37 *vs* 0.13, 2.71 *vs* 0.88, and 2.00 *vs* 0.61 respectively, with all p-values being less than 0.05.

**Figure 6:**
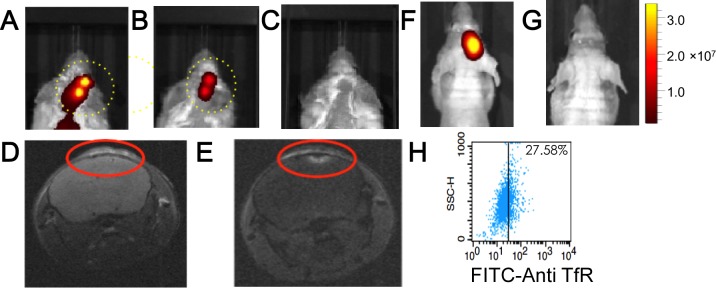
*In vivo* NIR-fluorescence imaging of platelets in control mice, and in mice bearing intra-cranial myeloma xenotransplants Cy7-fluorescence images of mice bearing intra-cranial xenotransplants that formed after injecting RPMI8226 cells. The mice (*n* = 3) were injected with Cy7/transferrin coupled KabC-platelets on day-5 and the Cy7-fluorescence imaged after 24-hours. Cy7-fluorescence images from two of these mice are shown in **A.** and **B.** show structured fluorescence from their crania. **C.** Cy7-fluorescence image of a control mouse without xenograft injected with Cy7/transferrin coupled KabC-platelets. The look up tables presented has units of radiant efficiency (photons.sec^−1^.cm^−2^.steradian^−1^) / μW.cm^2^. The mouse shown in Figure 6A was imaged by MRI on day-8; **D.** T1 image recorded before and, **E.** after injecting Magnevist in the tail vein. In a second control group, mice were injected with U87 cells transfected with RFP to allow for *in vivo* imaging of neuroglioblastoma xenotransplants. **F.** RFP fluorescence from a representative mouse in this group showing the locus of the neuroglioblastoma xenotransplants. **G.** corresponding Cy7-image of the same mouse injected with KabC-platelets coupled with transferrin and Cy7. Cy7 emission was not detected in the brain of this or other mice in the group. **H.** FACS analysis of U87 cells stained directly with a FITC-conjugated antibody against human transferrin receptor showing the majority of U87 cells express low levels of transferrin receptor.

Cy7-coupled platelets were cleared from the head regions of mice without intra-cranial tumors from 24 to 48-hours after their injection into mice, while other sites on the dorsal side were cleared from 48 to 96-hours (Figure 5A and 5B). The faster clearance of Cy7-coupled platelets from the head region of injected mice was exploited for high-contrast, NIR fluorescence imaging of intra-cranial xenotransplants that developed after injecting RPMI8226 cells. Myeloma xenotransplants grow rapidly in the intra-cranial cavity, a feature that allowed us to begin imaging studies only 5-days after the injection of RPMI8226 cells. KabC-platelets coupled with Cy7 and transferrin (test platelets) were injected into the tail veins of control mice ie those not injected with RPMI8226 cells (*n* = 3), and in test mice that had received intra-cranial injections of RPMI8226 cells (*n* = 3). Images of Cy7-fluorescence from the head region of test mice recorded 24-hours after injecting test platelets showed a structured fluorescence within the cranium (Figure 6A, 6B and Supplementary Figure 3C). The presence of Cy7-fluorescence on the lower dorsal sides 24-hours after injecting platelets in test mice (Figure 6A, 6B and Supplementary Figure 3C) did not interfere with our ability to use Cy7-fluorescence signals to identify the presence and location of intra-cranial myeloma transplants. This finding was supported by studies on control mice, which showed an absence of Cy7-fluorescence 24-hours after injecting test platelets (Figure 6C; Supplementary Figure 3A, 3B). Images from these control studies also support the view that test platelets do not engage in non-specific interactions with cells or structures in the head region of mice without myeloma xenotransplants. The findings from the *in vivo* Cy7-fluorescence imaging study was confirmed by MR-imaging of the cranium for the mouse shown in Figure 6A. The MRI study was conducted approximately 48-hours after injecting repurposed platelets. T1-images of the head region of the mouse were recorded immediately before and immediately after injecting 100 μL of the contrast-enhancing MRI probe (Magnevist, 0.1M). A qualitative comparison of the two images (Figure 6D and 6E, respectively) shows the Magnevist-labeled region in the cranium is similar to that identified 24-hours earlier from the Cy7-fluorescence (Figure 6A).

Taken together, *in vivo* imaging studies show injected KabC-platelets coupled with transferrin and Cy7 (test platelets) accumulate within RPMI8226 cell-derived myeloma xenotransplants. *in vivo* Cy7-fluorescence imaging showed accumulations of test platelets within early-stage intra-cranial myeloma transplants with diameters on the order of ∼1 mm. The bright Cy7-fluorescence recorded from these tumors arises in part from the efficient labeling of Cy7 molecules on the surface of intact repurposed platelets (diameter ∼2μm). Owing to differences in their size, repurposed platelets (φ ∼2000nm) can be surface-coupled with ∼100-fold more of a targeting protein or Cy7 compared to a nanoparticle (φ ∼200nm), and ∼1000-fold more of an internalized probe or cytotoxin.

**Figure 7:**
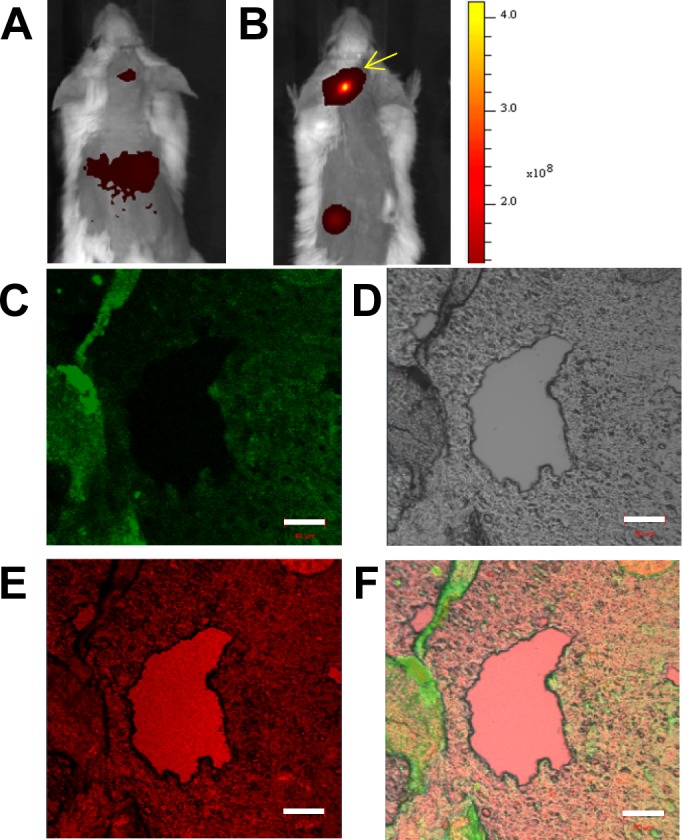
Immuno-histochemical imaging and analysis of RPMI8226 cell-derived xenotransplants Tumor slices excised from mice injected with KabC-platelets coupled with transferrin and chlorin e6. Measurement bars in all images is 50 μm: **A.** *in vivo* red-fluorescence image of a representative mouse bearing an intra-cranial xenotransplants that developed after injecting RPMI8226 cells and then injected with KabC-platelets loaded with chlorin e6. **B.** *in vivo* red-fluorescence image of a representative mouse bearing an intra-cranial xenotransplants that developed after injecting RPMI8226 cells and then injected with KabC-platelets coupled with transferrin and loaded with chlorin e6. **C.** Representative confocal fluorescence image of a FITC-labeled antibody directed against human transferrin receptor showing the distribution of RPMI8226 cells in the tumor slice. **D.** Bright-field image of the same tumor slice. **E.** Chlorin e6 fluorescence in the xenotransplant slice showing the distribution of repurposed platelets in blood vessels and tumor tissue. **F.** Overlay of the FITC, bright-field and red-fluorescence images showing infiltration of platelets within the xenotransplant.

We showed in figure 6C that control mice ie those without intra-cranial xenotransplants did not accumulate transferrin-coupled KabC platelets in the cranium, while control studies detailed in the following section also show platelets lacking transferrin do not accumulate to significant levels in the crania of mice bearing myeloma transplants (Figure 7A). A further control study was conducted to show the labeling of intra-cranial myeloma xenotransplants with KabC-platelets was dependent on interactions between transferrin molecules on the surface of the platelet and transferrin receptors on the surface of the tumor cell. A control group of mice (*n* = 3) were injected intra-cranially with RFP-transfected U87 cells that over time developed xenografts [23]. These mice were injected in their tail vein with KabC-platelets coupled with Cy7 and transferrin (test platelets). Neuroglioblastoma xenografts were clearly resolved from images of RFP-fluorescence that were recorded 120-hours after injecting the test platelets (Figure 6F). However, Cy7-fluorescence was not detected in the region around the RFP-labeled neuroglioblastoma xenotransplant (Figure 6G; Supplementary Figure 3D, 3E). Transferrin receptor expression levels on U87 cells were analyzed by FACS after incubating U87 cells for 30 minutes with a FITC-labeled anti-human transferrin receptor antibody. Approximately one quarter of the U87 cells showed a FITC signal beyond that of the autofluorescence signal the (Figure 6H). This study suggests that transferrin-coupled KabC-platelets bind to tumor cells that over-express the transferrin receptor where they are retained through multiple interactions. On the other hand, tumor cells that express low levels of the transferrin receptor, and other structures within the intra-cranial cavity do not show any significant binding to test platelets. With the exception of the liver and spleen, which are responsible for platelet clearance, we have not observed accumulations of test or control platelets in other tissues whose component cells express much lower levels of the transferrin receptor compared to RPMI8226 cells and K562 cells [13]. Another possibility to account for the increased specificity of test platelets for RPMI8226 cells in myeloma transplants would be if the human transferrin on the platelet had a higher affinity for the human transferrin receptor molecules on RPMI8226 cells compared to that for the murine transferrin receptor.

### Immuno-histochemical analysis of repurposed platelets in myeloma xenografts

Tissue slices prepared from intra-cranial xenotransplants were analyzed by immuno-histocytochemistry. The purpose of these studies was to further investigate the *in vivo* Cy7-imaging studies that showed KabC-platelets coupled with transferrin (test platelets) gain access to tumor microenvironments where they accumulate as a result of interactions between transferrin molecules on the platelets surface and receptor molecules on RPMI8226 cells. In this study, KabC-platelets were loaded with chlorin e6, a membrane permeable Cy5-like fluorescence probe that can also serve as a photodynamic therapy probe. Chlorin-e6 accumulates to a high level in the cytoplasm of KabC-platelets (Figure 1F). Chlorin e6-loaded KabC-platelets were divided into two fractions - one fraction was surface-coupled with transferrin (test) while the other (control) lacked transferrin. Control and test platelets were injected separately into the tail veins of mice (*n* = 3) that 5-days earlier had been injected intra-cranially with RPMI8226 cells. Chlorin-e6 fluorescence was recorded in live tumor-bearing mice using the Cy5 excitation and emission channel of the Caliper instrument. The chlorin e6 fluorescence was weak in the crania of tumor-bearing mice injected with control platelets (ie those lacking transferrin; Figure 7A; Supplementary Figure 3F, 3G). On the other hand, tumor-bearing mice injected with test KabC-platelets emitted fluorescence from their crania (Figure 7B, Supplementary Figure 3H, 3I). Tissue slices isolated from excised brains of mice injected with test platelets loaded with chlorin e6 were fixed using 4% paraformaldehyde and frozen-sliced. The fixed tissue section was further labeled with a FITC-labeled antibody directed against the transferrin receptor. After extensive washing, regions from a representative slice of the multiple myeloma isolated from the mouse shown in Figure 7B were observed visually, and representative images of the FITC channel (green; transferrin receptor; Figure 7C) and chlorin e6 channel (pink; platelets; Figure 7E), and bright-field (Figure 7D) were recorded on the Zeiss 700 confocal microscope. The three images (FITC and chlorin e6 fluorescence, and bright-field) of the tumor slice were merged using embedded software in the Zeiss instrument (Figure 7F). FITC-fluorescence, which serves as a measure of RPMI8826 cells, was distributed throughout the tumor tissue (Figure 7C). Chlorin-e6 fluorescence (pink) was strongest in the blood vessels (Figure 7E), although significant fluorescence was also evident in the tumor tissue (Figure 7F). The merged image of the tumor slice reveals shows considerable overlap in the distributions of the transferrin receptor (FITC) and test platelets (chlorin e6). On the basis of analyses of results from the immunofluorescence-histocytochemical analysis and Cy7-fluorescence images of organs excised from tumor-bearing mice (Figure 5C), we conclude that KabC-platelets surface-coupled with transferrin are present within tumor microenvironments where they are retained by specific interactions between transferrin on the platelet surface and high levels of transferrin receptors on RPMI8826 cells.

## DISCUSSION

We have shown that human platelets can be repurposed as vehicles for efficient *in vitro* and *in vivo* targeting and imaging of myeloma xenotransplants by coupling targeting proteins and detection probes to their surface. The effectiveness of repurposed platelets as vehicles for tumor-targeting and imaging is a consequence of several unique features that include: (a), privileged and deep access to tumor microenvironments; (b), a long period of circulation of up to 9-days, which increases their chance of encountering a tumor cell compared to most nanoparticle vehicles that have circulation times on the order of 3∼9 hours [7]; (c), greater volume and surface area that allows platelets to transport a larger cargo of cytotoxins, detection probes and surface-borne targeting proteins compared to a nanoparticle [6]; (d), repurposed platelets are cleared naturally by Kupfer cells in the liver and spleen, which reduce non-specific accumulation of labeled platelets at off-target sites. The short circulation time of nanoparticles of 3∼5 hours [6] may help to increase signal contrast of a targeted tissue within a few hours of their injection into an animal. This advantage however, must be weighed against two very negative and application-limiting effects of using vehicles with short circulation times. First, the short period of circulation reduces the number of molecular encounters between the injected nanoparticles and their target cells *ie* most nanoparticles will be cleared by macrophages *via* endocytosis or *via* the liver before they encounter the target. Consequently, effective labeling of a tumor requires one to inject large numbers of nanoparticles, which also increases the risk of off-targeting. Second, in the case of nanoparticles loaded with cytotoxins the sudden appearance of high levels of the cytotoxin in the liver may overwhelm the detoxification capacity of the liver and result in liver damage. The longer circulation of platelets (9-days) on the other hand, would increase their interactions with targeted cells, while their gradual and exclusive clearance by the liver and spleen should help to reduce cytoxin-mediated damage to these organs. The application of longer-circulating, repurposed platelets as tumor-targeting vehicles would also help to reduce the number of repeat injections in a cancer treatment.

Inhibiting platelet-aggregation is an essential step in repurposing human platelets as living vehicles for tumor targeting and imaging. Platelet-aggregation is blocked by passive loading of KabC, or cytochalasin D [24] in the cytoplasm where they inhibit actin polymerization and membrane protrusions associated with platelet-aggregation. We have also used salicylic acid to inhibit platelet-activation and aggregation [25]. Platelets rendered aggregation-incompetent by treatment with KabC can be loaded passively with a variety of membrane permeable drugs and detection probes, including chlorin e6, CFDA and EPI. The accumulation and retention of these molecules in the cytosol was shown by confocal fluorescence imaging and FACS. Interestingly, chlorin e6 may be used as a Cy5-like detection probe as was shown in this study, and as photodynamic therapy probe [18]. The reactions used to repurpose human platelets for targeted imaging of myeloma were optimized using FACS and fluorescence microscopy - repurposed platelets configured with KabC, EPI, transferrin and Cy7 were prepared within an hour of receiving outdated human platelets. Tumors that developed after injecting RPMI8226 cells accumulated injected KabC-platelets that had been surface-coupled with Cy7 and transferrin. These platelets resulted in a strong Cy7-fluorescence that was used for *in vivo* imaging of intra-cranial tumors of ∼1 mm in diameter. The protocols we have developed to repurpose human platelets as vehicles for *in vivo* targeting of myeloma xenotransplants can be modified to accommodate a large range of targeting ligands and antibodies, and therapeutic agents and detection probes. For example, we have recently shown that KabC-platelets can be configured with a plurality of detection probes, including NIR-fluorophores, Magnevist and iron oxide nanoparticles for MR-imaging, and therapeutic proteins. Since platelets are found naturally at sites of inflammation and tissue damage, their repurposing with additional targeting, and therapeutic proteins could be used to augment innate immune responses to a disease condition, including those associated with cardiovascular disease and neurodegenerative disorders.

Looking forward, recent studies from our group indicate additional applications for repurposed platelets in translational and precision medicine. These findings include demonstrations that platelets coupled with an antibody against the protein death ligand-1 bind selectively to tumor cells that express PDL-1 [26]. Platelets have also been configured with a plurality of antibodies that allow for tumor-targeting and T-cell activation.

## MATERIALS AND METHODS

RPMI8226 multiple myeloma and K562 leukemia cells were obtained from the cell bank of the Chinese Academy of Sciences. Human platelets were obtained from the Chinese Red Cross within 5 days of drawing blood. Platelets were stored in citrate saline (0.006M tri-sodium citrate/ 0.154M NaCl, pH 6.8) with 5% bovine serum albumin at a density of 3-5×108 platelets/ml. After centrifugation, platelet pellets were re-suspended in a modified Hanks' buffered salt solution (mHBSS; 0.17M NaCl/ 6.7mM KCl/ 1.0mM MgSO4/ 0.5mM K2HPO4/ 2.8mM Na2HPO4 / 13.8mM dextrose, pH to 7.2 with 1.4% NaHCO3) for *in vitro* studies, or in normal saline for *in vivo* studies. KabC and TMR-KabC were prepared according to our earlier publications [10-12]. Human transferrin, EPI, carboxyfluorescein diacetate (CFDA), chlorin E6 (CE6), Phenyl-dimaleimide, maleimide-benzoic acid succinimide ester and iminothiolane were purchased from Sigma. Cy5-NHS and Cy7-NHS were both purchased from GE Healthcare.

### Transferrin conjugates

PDM-transferrin was prepared by reacting 25 μM PBS solution of transferrin with 250 μM of 2-iminothiolane to generate thiol groups on the protein surface. After passage over a PD-10 column in PBS the protein fraction was treated with PDM to 250 μM and after a 2-hour incubation at 20°C the sample was applied to a second PD-10 column to remove excess crosslinker. The thiol-reactive conjugate was stored in 100 μL aliquots at −20°C. A transferrin conjugate harboring both Cy5 and maleimide-benzoic acid succinimide ester (MBS) was prepared by incubating transferrin (5mg/ml) in PBS with Cy5-NHS (0.2mg/ml) and MBS (0.1 mg/ml) delivered from DMF stock solutions. After a 2-hour incubation unbound reactants were removed by PD-10 chromatography and the protein conjugate characterized by absorption spectroscopy and SDS-PAGE and stored at −20°C in 100μL aliquots. The MBS/Cy7-conjugate of transferrin was prepared using the same protocol. Protocols used to couple transferrin conjugates to thiolated platelets are described in the Results section.

### Confocal fluorescence microscopy

Fluorescence images of probes loaded into the cytoplasm or on the surface of platelets, and in mixtures of platelets with surface-attached RPMI 8226 or K562 cells, within immuno-histocytochemical stained frozen myeloma xenograft tissue sections were carried out using a Zeiss 700 instrument with embedded software that allows for excitation of fluorescent probes (CFDA, FITC, EPI, TMR, or Cy5) at 488nm, 555nm and 639nm, and collection of the fluorescence emission from each probe was using a computer-selected filter.

### FACS

A Becton and Dickenson FACS Calibur flow cytometer was used to sort and quantify labeled populations of free platelets and RPMI8226 or K562 cells. These studies recorded 10,000 events for each sample using the fluorescence of CFDA, FITC, EPI, TMR, Cy5 or CE6 that were detected using FL1, FL2, FL3 or FL4 channel respectively. The FACS data was analyzed with FlowJo V3.2 (Tree Star, Inc.) and represented as the percentage of labeled population.

### Electron microscopy

Platelets were prepared for SEM and TEM-imaging using an established protocol [27] in a Hitachi 450 SEM and a FEI Tecnai G2 Spirit Bio TWIN TEM. Suspensions of platelets were fixed chemically by adding an equal volume of 0.1% glutaraldehyde for 15-minutes followed by centrifugation and re-suspension of the pellets in 3% glutaraldehyde. The platelet samples were allowed to settle and adhere to glass fragments that were pre-coated with poly-lysine. The glass fragments were rinsed with distilled water and dried at the critical-point.

### Mice

Analgesic and tranquilizing drugs were used to minimize discomfort and pain to animals during injections of RPMI8226 cells. NOD/SCID mice are euthanatized by cervical dislocation after anesthetization with a 0.016 mL/g (body weight) solution of 2.5% Avertin that was injected intra-peritoneal. Alternatively, death was brought about by CO_2_ anesthesia followed by decapitation. Experiments on living mice were carried out according to established protocols developed at SouthEast University, and were consistent with an animal protocol used by GM at UC-Berkeley.

### NIR-fluorescence imaging

RPMI8226 cells (1×10^7^ cells in 100μl of buffer) were injected under the skin on the backs of NOD/SCID mice. In other studies RPMI8226 cells (1×10^6^ cells in 10μl) were injected through an opening in the skull of NOD/SCID mice [14-17]. A minimum number of mice were used for these proof of practice studies, and typically 3 in each data group. NIR fluorescence imaging of live mice and their excised organs was carried out using a Caliper IVIS Spectrum Imaging System. The instrument was used to record the NIR emission spectra of Cy5 and Cy7 loaded in platelets within live mice, or from their excised organs. The NIR fluorescence images shown in this study were processed using software resident in the IVIS machine. Prior to *in vivo* imaging, fur in the vicinity of the myeloma xenotransplant was removed from the mice by shaving or defoliation.

## SUPPLEMENTARY MATERIAL FIGURES


