# Supplementary material for: Human platelets repurposed as vehicles for in vivo imaging of myeloma xenotransplants
Source: Oncotarget. 2016 Mar 31;7(16):21076–90. doi: 10.18632/oncotarget.8517 (PMC5008270; doi:10.18632/oncotarget.8517)
Supplement: Supplementary file 1 [file oncotarget-07-21076-s001.pdf]

Human platelets repurposed as vehicles for *in vivo* imaging of myeloma xenotransplants

Supplementary Material

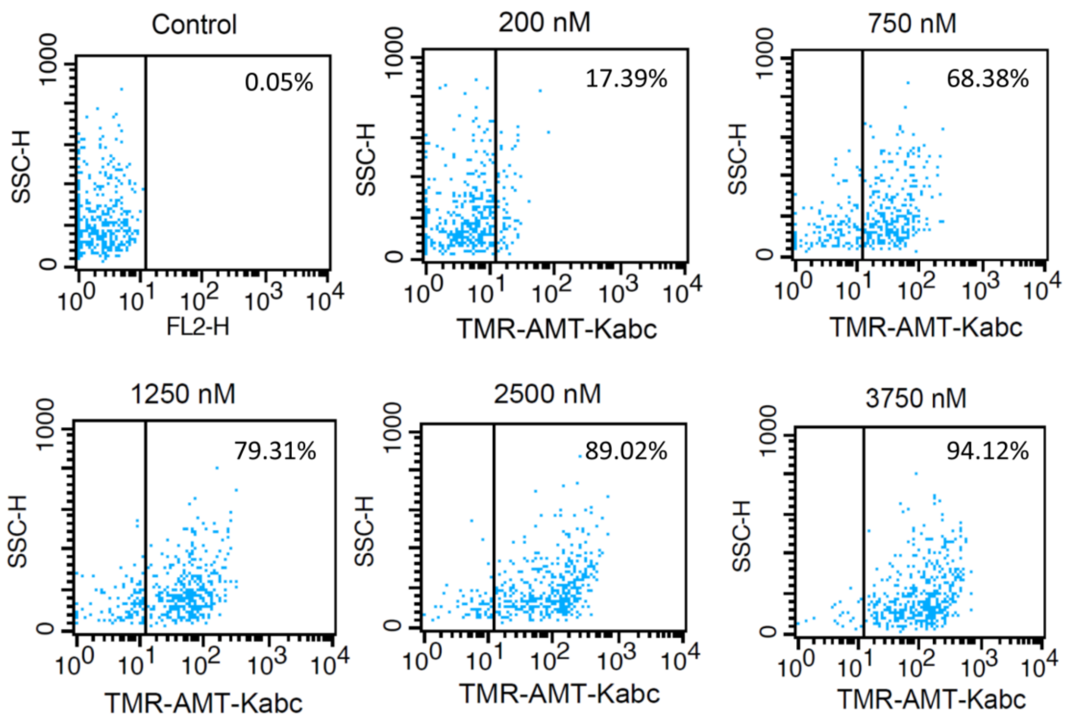

Supplementary Figure 1:

FACS analysis of TMR-KabC loading of human platelets.

FACS analysis of TMR-KabC loading of purified human platelets 24 hours after their incubating with defined concentrations of TMR-KabC with 10^8 platelets in 1 ml of platelet buffer.

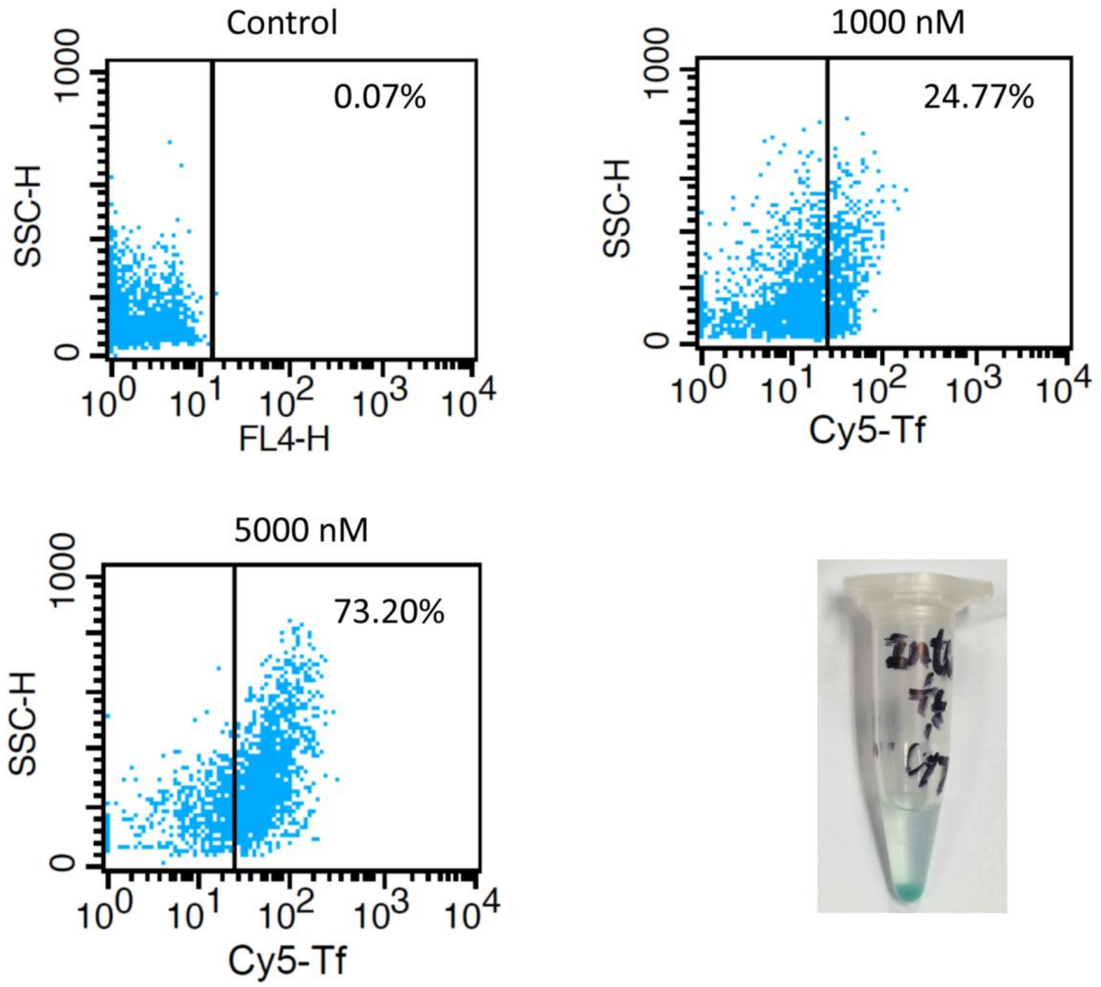

Supplementary Figure 2:

FACS analysis of the chemical-coupling of PDM/Cy5-transferrin to thiol-containing KabC-platelets.

FACS analysis of the optimized loading condition for Cy5/PDM transferrin coupling to thiol-containing KabC-platelets. The photograph on the bottom right shows an Eppendorf tube containing a suspension of KabC-platelets coupled on their surface with Cy7-NHS that had been allowed to settle by gravity. Sedimented platelets are heavily labeled with Cy7 as can be seen by the strong absorption of far red light by Cy7 molecules

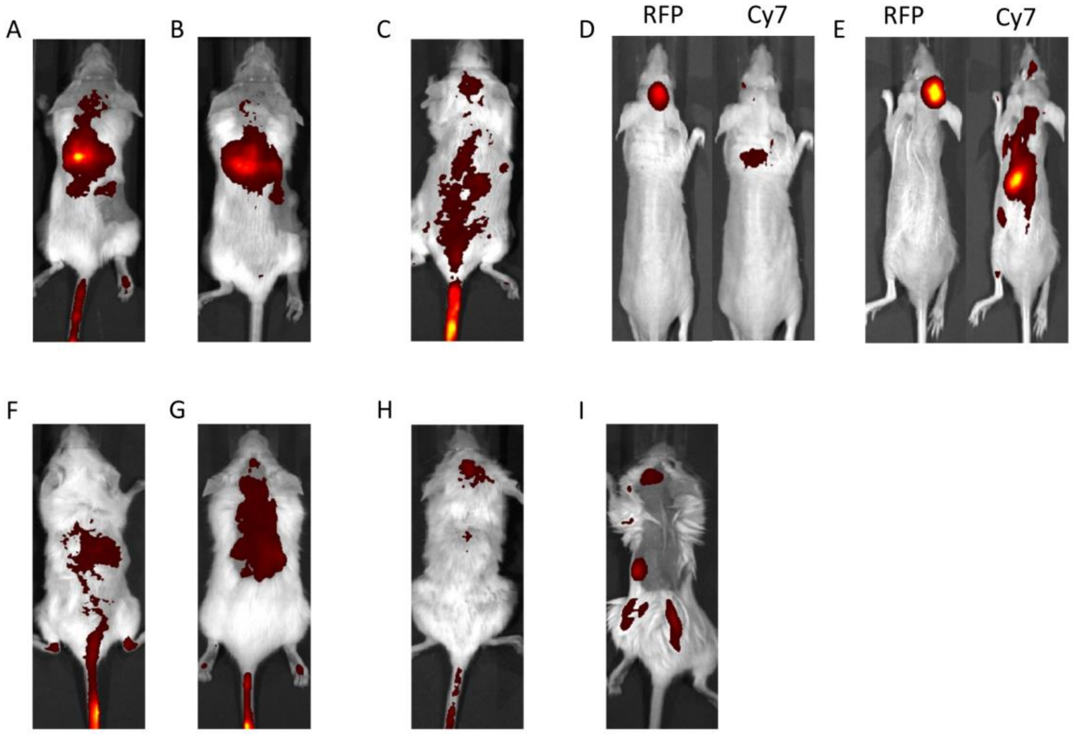

Supplementary Figure 3 A-I

Supplementary Figure 3:

Proof-of-practice, in vivo imaging of repurposed platelets in myeloma xenotransplants

A, B. KabC-platelets coupled with Cy7 and transferrin were injected into the tail veins of two control mice without RPMI8226 cells. **C.** KabC-platelets coupled with Cy7 and transferrin were injected into one of the three mice that 5-days earlier had been injected intra-cranially with RPMI8226 cells. **D, E.** KabC-platelets coupled with Cy7 and transferrin were injected into the tail veins of two mice that had been injected intra-cranially with RFP-transfected U87 cells. **F, G.** Chlorin e6-loaded KabC-platelets lacked transferrin were injected into the tail veins of two mice that had been injected intra-cranially with RPMI8226 cells. **H, I.** Chlorin-e6 loaded KabC-platelets surface-coupled with transferrin were injected into the tail veins of two mice that had been injected intra-cranially with RPMI8226 cells.
